# Mediation and moderation of genetic risk of obesity through eating behaviours in two UK cohorts

**DOI:** 10.1093/ije/dyad092

**Published:** 2023-07-06

**Authors:** Shahina Begum, Eleanor C Hinton, Zoi Toumpakari, Timothy M Frayling, Laura Howe, Laura Johnson, Natalia Lawrence

**Affiliations:** Department of Psychology, College of Life and Environmental Sciences, University of Exeter, Exeter, UK; NIHR Bristol BRC Nutrition Theme, University Hospitals Bristol Education & Research Centre, University of Bristol, Bristol, UK; Centre for Exercise, Nutrition and Health Sciences, School for Policy Studies, University of Bristol, Bristol, UK; Genetics of Complex Traits, University of Exeter Medical School, Exeter, UK; Population Health Sciences, Bristol Medical School/MRC Integrative Epidemiology Unit, University of Bristol, Bristol, UK; Centre for Exercise, Nutrition and Health Sciences, School for Policy Studies, University of Bristol, Bristol, UK; Department of Psychology, College of Life and Environmental Sciences, University of Exeter, Exeter, UK

**Keywords:** Obesity, BMI, ALSPAC, eating behaviour, dietary restraint, disinhibition, hunger, genes, genetic risk score, appetite

## Abstract

**Background:**

The mechanisms underlying genetic predisposition to higher body mass index (BMI) remain unclear.

**Methods:**

We hypothesized that the relationship between BMI-genetic risk score (BMI-GRS) and BMI was mediated via disinhibition, emotional eating and hunger, and moderated by flexible (but not rigid) restraint within two UK cohorts: the Genetics of Appetite Study (GATE) (*n* = 2101, 2010–16) and the Avon Longitudinal Study of Parents and Children (ALSPAC) (*n* = 1679, 2014–18). Eating behaviour was measured by the Adult Eating Behaviour Questionnaire and Three-Factor Eating Questionaire-51.

**Results:**

The association between BMI-GRS and BMI were partially mediated by habitual, emotional and situational disinhibition in the GATE/ALSPAC meta-mediation [standardized beta_indirect_ 0.04, 95% confidence interval (CI) 0.02–0.06; 0.03, 0.01–0.04; 0.03, 0.01–0.04, respectively] external hunger and internal hunger in the GATE study (0.02, 0.01–0.03; 0.01, 0.001–0.02, respectively). There was evidence of mediation by emotional over/undereating and hunger in the ALSPAC study (0.02, 0.01–0.03; 0.01, 0.001–0.02; 0.01, 0.002–0.01, respectively). Rigid or flexible restraint did not moderate the direct association between BMI-GRS and BMI, but high flexible restraint moderated the effect of disinhibition subscales on BMI (reduction of the indirect mediation by -5% to -11% in GATE/ALSPAC) and external hunger (-5%) in GATE. High rigid restraint reduced the mediation via disinhibition subscales in GATE/ALSPAC (-4% to -11%) and external hunger (-3%) in GATE.

**Conclusions:**

Genetic predisposition to a higher BMI was partly explained by disinhibition and hunger in two large cohorts. Flexible/rigid restraint may play an important role in moderating the impact of predisposition to higher BMI.

Key Messages

- Increased genetic predisposition to body mass index (BMI) was associated with higher BMI in adults in two UK cohorts.
- Mediation analyses found that predisposition to higher BMI was partly explained by eating behaviours (e.g. increased disinhibition, emotional eating and susceptibility to hunger).
- Mediating eating behaviours measured by Adult Eating Behaviour questionnaire in adulthood were different to those published in childhood studies (e.g. satiety responsiveness, food responsiveness and enjoyment of food in children in contrast to emotional eating and hunger in adults).
- Rigid and flexible restraint may ‘counter’ predisposition to higher BMI via disinhibition and external hunger.

## Introduction

Obesity is a leading global public health issue, associated with approximately 5% of premature mortality.^1^ The rising prevalence of obesity has been largely attributed to an obesogenic environment and individual genetic variation.^2^^,^^3^ Genetic variation explains 40–70% of differences in body mass index (BMI).^4^ Previous heritability estimates divided variation of a trait into genetic and environmental contributions, but recent genome-wide association studies (GWAS) have identified over 900 single nucleotide variants (SNVs) that are associated with individual weight, indicating interindividual differences in the susceptibility to obesity.^5^^,^^6^ Individually these account for a small proportion of the variance in BMI; thus subsequent studies aggregated the effects of variants to evaluate the influence of all SNVs simultaneously and to easily assess individual risk to obesity using a single score.

Whiereas precise mechanisms underlying risk variants associated with obesity are being explored, many BMI candidate loci are enriched in genes thought to modulate reward, hunger and satiety pathways within the central nervous system.^2^^,^^5–10^ Early twin studies provide strong heritability estimates for genetic influence on eating behaviours across all ages, partly explaining increases in BMI in children and adults.^11^ Given the subsequent development of the behavioural susceptibility theory (eating behaviour as a key behavioural pathway in which individuals are predisposed to obesity^2^^,^^3^) and GWAS discoveries, several studies measuring appetite and food intake psychometrically have reported mediation through eating behaviours, in particular via uncontrolled eating,^12^^,^^13^ and the related traits of disinhibition and hunger.^13–17^ Only one study has examined the type of uncontrolled eating in detail, suggesting habitual disinhibition may mediate genetic risk more than the other traits examined (e.g. emotional/situational disinhibited eating).^18^ Dietary restraint has also been explored as a mediator and moderator.^15^^,^^18^ The type of restraint (e.g. flexible versus rigid control over food intake) could influence the effect of genetic susceptibility to obesity via uncontrolled eating,^19–23^ but this has not been investigated. We used detailed measures of disinhibition, hunger and restraint in two large population-based cohorts with 600+ variant BMI-genetic risk scores (BMI-GRS) to investigate the established pathway between BMI-GRS and BMI through eating behaviours. Specifically, we used mediation analyses to decompose the influence of BMI-GRS on BMI directly, and indirectly via disinhibition in response to habitual and situational cues and, to a lesser extent, emotional disinhibition, emotional over-and-under eating and hunger in response to internal and external triggers. We expected flexible (but not rigid) restraint to moderate the association between BMI genetic risk and BMI, as well as attenuate the indirect influence of the aforementioned eating behaviours.

## Methods

### Settings/participants

Data used in this study were from two UK cohorts, the EXETER 10 000 Genetics of Appetite sub-study (GATE) and the children of the Avon Longitudinal Study of Parents and Children (ALSPAC).

#### The GATE study

**Figure 1.**
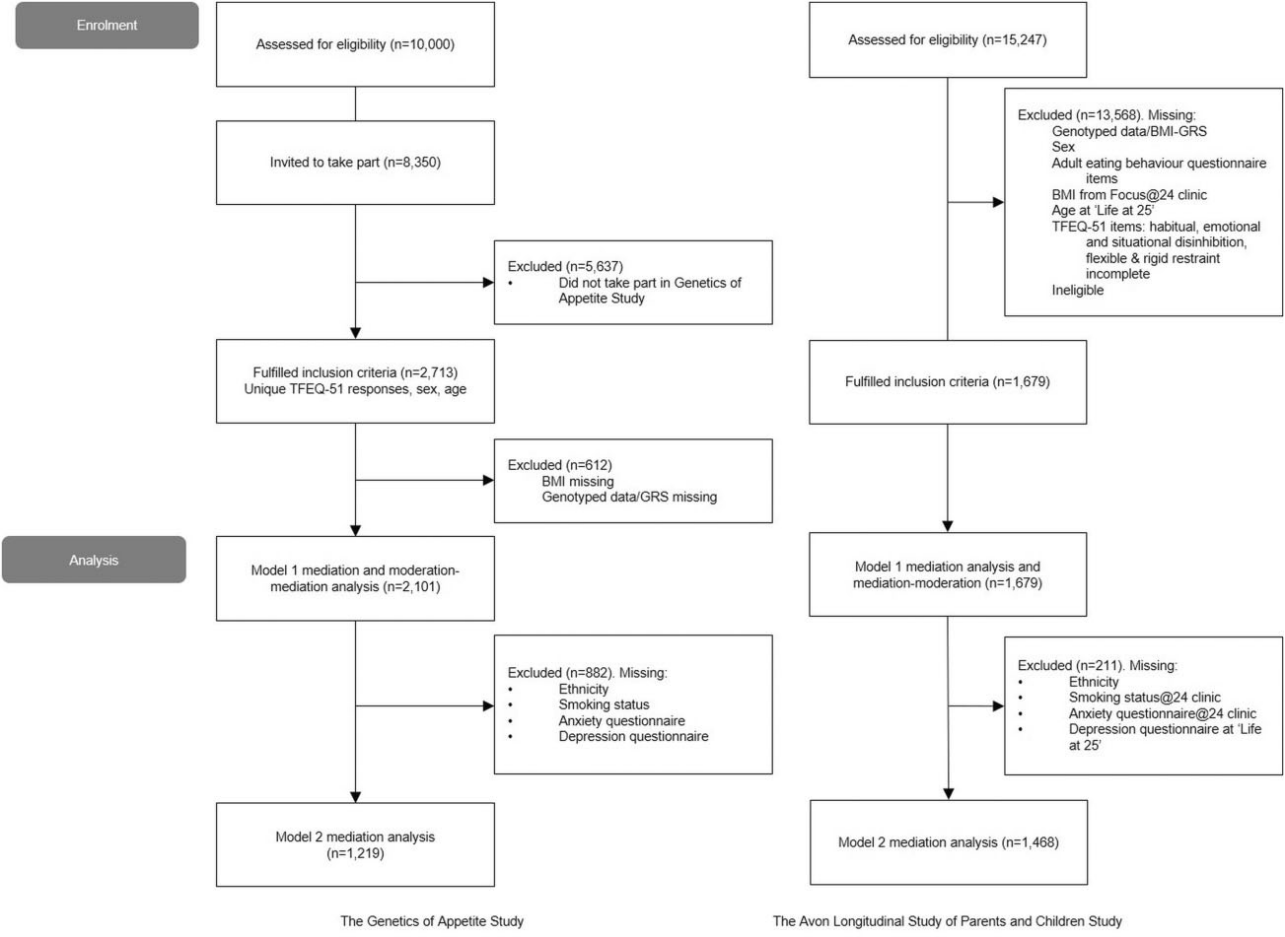
Selection of participants. BMI, body mass index; BMI-GRS, body mass index-genetic risk score; TFEQ-51, Three-Factor Eating Questionnaire-51. The Genetics of Appetite study included people who participated in the Exeter 10 000 project (EXTEND) organised by NIHR Exeter Clinical Research Facility (CRF) and its extension study, Genetics of Appetite (GATE). By distributing invitations via the CRF annual newsletters as well as individual letters to 4549 people (invited between December 2012 and May 2013) and 3794 people (invited between June 2013 and April 2017), we were able to collect 2715 participants in total. The inclusion criteria for participants were: (i) ages 18 and over at the time of application; and (ii) possess a permanent address within 25 miles of Exeter; 2713 respondents fulfilled inclusion criteria, of whom 1432 individuals responded to the first Three-Factor Eating Questionnaire (TFEQ). The invitation was sent out once more, this time 1813 individuals responded, of whom 531 had responded to the first questionnaire. For participants who responded twice, responses from their first TFEQ were used in this study (*n* = 2713). The Avon Longitudinal Study of Parents and Children cohort is a birth cohort of 14 451 pregnancies from the south-west of England, UK, between 1991 and 1992, increasing to 15 247 pregnancies by the age of 18 years.^25^^,^^26^ The recruitment and sample sizes of the ALSPAC cohort are complex due to the repeat attempts to bolster the initial sample size. Pregnant women who resided within three health districts within Avon and were estimated to deliver between 1 April 1991 and 31 December 1993 were eligible. There are now three key cohorts within ALSPAC: the mothers, the first generation of offspring (‘The Children of the 90s’) and the second generation of children (ALSPAC-G2). Participants in this study included offspring of the mothers in the original cohort e.g. the children born in the 90s. Data were used from participants with DNA samples who took part in the Focus@24 clinic (June 2015 to October 2017) and self-completed questionnaire assessments ‘Me at 23+’ at age 23 years (IQR 23 to 24) which included the AEBQ and ‘Life at 25+’ at age 25 years (IQR 25 to 26) which included the TFEQ^27^

Healthy men and women, age >18 years and resident within 25 miles of Exeter (UK) were recruited for the EXETER 10 000 study between 2010 and 2016. The wider study explored the development of common diseases.^24^ Participants completed a baseline clinical visit at median age 59 years [interquartile range (IQR) 47 to 66]. Data were collected about their health, lifestyle and body measurements, and blood and urine samples were taken. Participants included in the present study were those who took part in the GATE sub-study for whom genotype data for 605 BMI-related single nucleotide polymorphisms (SNPs) from a 2015 GWAS meta-analysis of BMI, BMI at baseline visit (measured between January 2010 and November 2016) and Three-Factor Eating Questionnaire-51 (TFEQ-51) responses [collected between December 2012 to April 2017, median age 62 years (IQR 51 to 69)] were available.^5^ TFEQ-51 items were collected twice for 531 individuals who repeated the questionnaire during a second recruitment drive. The first TFEQ-51 score was used as the mediator in the present study. There was a median 1.7 (0.92 to 2.78 interquartile range) years between BMI and first TFEQ measurement. No additional exclusion criteria were enforced and those with comorbidities were included. Figure 1 shows a diagram of the participant selection.

#### The ALSPAC study

ALSPAC is a birth cohort of 14 451 pregnancies from Bristol (UK) between 1991 and 1992, increasing to 15 247 pregnancies by the age of 18 years.^25^^,^^26^ Selected subgroups have been regularly followed up using more than 60 questionnaires and 10 clinical assessment visits. Participants in the current study included adult offspring of the mothers in the original cohort. Data were used from participants with DNA samples who took part in the Focus@24 clinic (June 2015 to October 2017) and self-completed questionnaire assessments ‘Me at 23+’ at age 23 years (IQR 23 to 24) which included the Adult Eating Behaviour Questionnaire (AEBQ) and ‘Life at 25+’ at age 25 years (IQR 25 to 26) which included the TFEQ-51^27^ (Figure 1).

### Measurements used in this study

#### Genotyping and BMI-genetic risk score

Genotyping was performed using the Illumina Infinium Global Screening Array in both cohorts. Imputation of genotypes was performed using the Haplotype Reference Consortium (HRC) imputation reference panel. Genotyping in ALSPAC has been described in detail previously.^25^^,^^28^^,^^29^ The genetic risk score was calculated by the sum of the total number of risk alleles at 605 (GATE) and 934 (ALSPAC) of the 941 BMI-associated loci reported in GWAS studies published in 2015 and 2018, respectively, weighted by their estimated effect sizes.^5^^,^^6^ Higher scores indicate a greater genetic predisposition to obesity. BMI-GRS were internally standardized z scores for ease of comparison across cohorts.

#### Eating behaviour

Eating behaviours were collected using items from the TFEQ-51 in both cohorts and the AEBQ in ALSPAC only. The TFEQ-51 is a validated questionnaire used to assess three dimensions of eating behaviour: cognitive restraint, disinhibition and susceptibility to hunger.^30^ Cognitive restraint is the intention to restrict food intake to lose or control body weight. Disinhibition is the overconsumption of food associated with a loss of control. Susceptibility to hunger (or perceived hunger) represents food intake in response to feelings and perceptions of hunger.^31^ Subscales include rigid restraint (an ‘all or nothing’ dieting approach), flexible restraint (a more lenient approach to controlling food intake e.g. ‘When I have eaten my quota of calories I am usually good about not eating any more’),^32^ disinhibition triggered by habitual, emotional and situational cues, and internal/external locus of hunger (feelings of hunger that are interpreted and regulated internally or external cues, respectively).^33^ Higher scores indicate a greater prominence of these traits; the minimum-maximum range and Cronbach alpha are available in Supplementary Table S1 (available as Supplementary data at *IJE* online). The AEBQ assessed four food-approach traits: food responsiveness (e.g. ‘When I see or smell food that I like, it makes me want to eat’), emotional overeating (e.g. ‘I eat more when I am upset’), enjoyment of food, and hunger (measure of physical hunger e.g. stomach rumbles). Four food-avoidance appetitive traits were also measured: satiety responsiveness (e.g. ‘I get full up easily’), slowness in eating, food fussiness and emotional undereating.^34^^,^^35^ The GATE study participants answered all TFEQ-51 items, but not the AEBQ. The ALSPAC study collected TFEQ-51 disinhibition and cognitive restraint subscales within the Life at 25+ questionnaire. TGEQ-51 hunger items were excluded due to overlap with ‘hunger’ scales already collected in the earlier AEBQ within the ‘Me at 23+’ questionnaire.

#### Confounders

Additional variables included in the adjusted analyses include smoking, depression (Patient-Health Questionnaire and a modified Mood and Feelings Questionnaire),^36^^,^^37^ anxiety (Generalised Anxiety Disorder-7),^38^ sex, age and ethnicity. These were collected during the baseline clinic visit and online questionnaire within the GATE study (median age 59 years and 62 years, respectively) and questionnaires at 23 to 25 years within the ALPAC cohort (Supplementary Table S2, available as Supplementary data at *IJE* online for details).

#### BMI (outcome)

As part of the original EXETER 10 000 study requirement, all participants attended a baseline appointment with a clinician at the Royal Devon and Exeter Hospital, where height and weight were taken and were used to calculate BMI (kg/m^2^). Standing height was measured using a Harpenden wall-mounted stadiometer. Weight was measured using a Tanita TBF-401A electronic body composition scale. Measurements were taken from participants in the ALSPAC cohort during the Focus@24 clinic using a Harpenden wall-mounted stadiometer and Tanita TBF-401A electronic body composition scales (or electronic bathroom scales).^25^

### Statistical analyses

Variables were described using means (standard deviation), median (IQR) or frequency (proportion). Mediation of the genetic risk to obesity and BMI was assessed using structural equation models (SEM). Compared with the individual linear regressions (e.g. Baron and Kenny conditions and the Sobel test^39^), using SEM enabled more power to simultaneously detect indirect effects, calculate accurate standard errors and assess interactions. Two SEM were developed for each eating behaviour. Model 1 included age and sex. Model 2 additionally included current smoking status, depression, anxiety and ethnicity (Supplementary Figure S1, available as Supplementary data at *IJE* online). The results of Model 2 will be presented as the main findings. SEM estimated the direct (‘c’) and indirect (the pathway from BMI-GRS to the BMI through the eating behaviour, ‘a’ * ‘b’) and total effect (sum of the direct and indirect effects) of BMI-GRS on BMI using simultaneous linear regressions and bootstrapping (x 1000). We conducted a linear model with BMI-GRS (independent variable) which assumed an additive genetic inheritance model (rather than dominant or recessive). Each eating behaviour from TFEQ-51 and AEBQ was tested separately, and not pooled together. Assumptions of a linear relationship between eating behaviours and BMI-GRS were assessed using a likelihood ratio test. Normality of residuals was evaluated for TFEQ-51 and AEBQ models via visual inspection of residual histograms and Q-Q plots. Mediation results for common subscales (i.e. habitual, emotional and situational disinhibition) from GATE and ALSPAC were meta-analysed to increase sample size and precision of estimates. Sensitivity analyses included testing measurement error of the mediator (eating behaviour) by fixing reliability to 0.8 and intermediate confounding (for depression and anxiety). Mediation by restraint was not tested as we were interested in the role of restraint as a moderator.

First, we tested rigid and flexible restraint interaction on the direct relationship between BMI-GRS and BMI. Second, we tested whether rigid and flexible restraint moderated the effect of eating behaviour (mediator) on BMI (outcome), also known as moderated-mediation (model depicted in Supplementary Figure S2, available as Supplementary data at *IJE* online).^40^ Moderated-mediation was tested by including a continuous flexible or rigid restraint interaction variable (separately) in the model. Mediation models were stratified into ‘high’ and ‘low’ flexible and rigid restraint (a score of ≤3 was low restraint, >3 was high restraint as recommended^31^^,^^41^) to illustrate the moderating effects of flexible and rigid restraint on the overall mediation in a binary fashion. Data were analysed using Stata software, version 16.0 (StataCorp, College Station, TX). There was no formal adjustment for multiple comparisons because of the exploratory nature of the study. Missing data were not imputed; however, differences between participants included in Model 1 and participants who were excluded due to missing data were assessed via t tests and chi square analyses. Last, analyses were treated as cross-sectional and ‘habitual’ (representative of the entire period of the study).

## Results

### Eating behaviour as mediators of genetic risk of obesity

**Figure 2.**
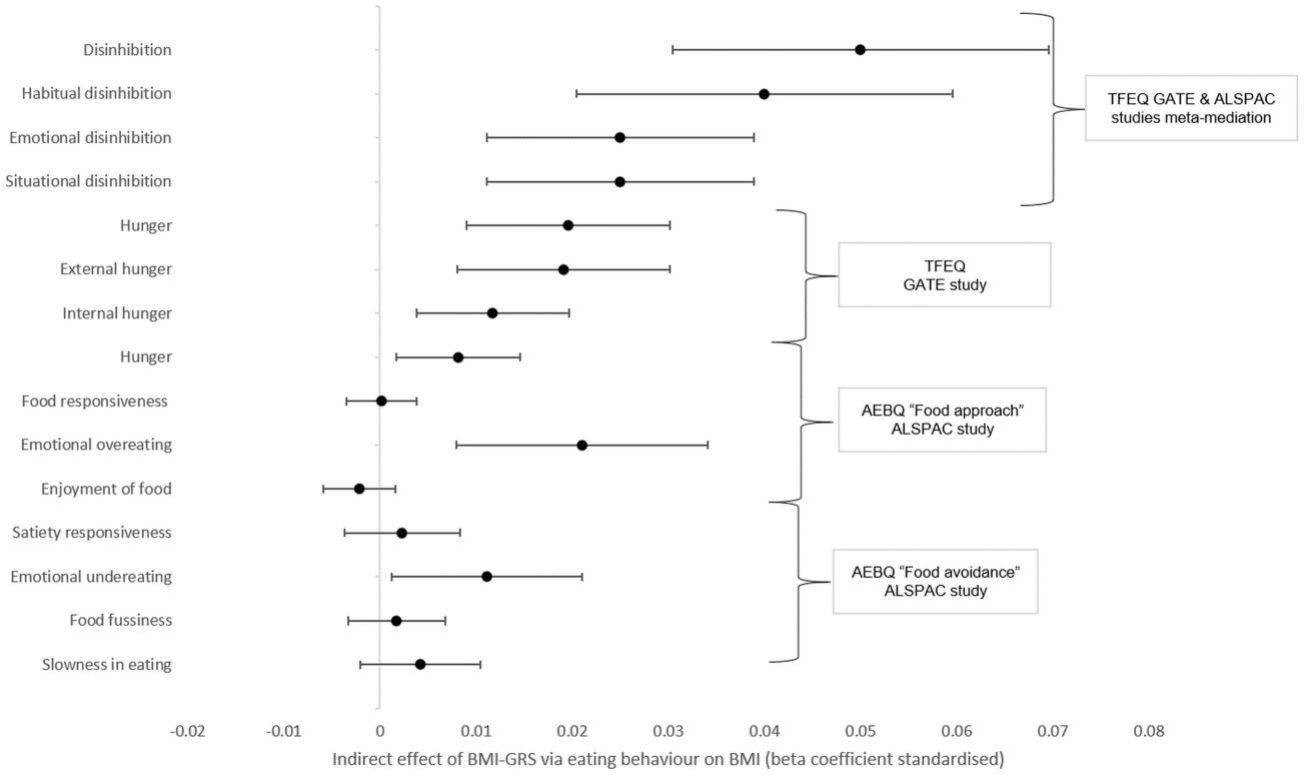
Indirect effect of body mass index genetic risk score mediated by eating behaviour (standardized, Model 2). AEBQ, Adult Eating Behaviour Questionnaire; ALSPAC, Avon Longitudinal Study of Parents and Children; BMI, body mass index; BMI-GRS, body mass index genetic risk score; GATE, Genetics of Appetite study; TFEQ, Three Factor Eating Questionnaire. The indirect effect was the product of a and b (a * b) which represents the mediation effect of the eating behaviour traits on the association between BMI-GRS and BMI. Model 2: smoking, ethnicity, depression, anxiety, sex, age were controlled for in relationship a; ethnicity, depression, anxiety, sex and age controlled for in relationship b (see Supplementary Figure S3, available as Supplementary data at *IJE* online for Model 1, adjusted for sex and age only)

All eating behaviours measured by the TFEQ-51 and three of the eight AEBQ eating behaviours partly mediated the association between BMI-GRS and BMI (Table 1 reports the total, direct, indirect, pathways ‘a’ and ‘b’, Figure 2 illustrates standardized indirect estimates only). Specifically, disinhibition, and subscales habitual disinhibition, emotional disinhibition and situational disinhibition were partial mediators in the GATE and ALSPAC meta-mediation. Hunger and hunger subscales (external hunger and internal hunger) were partial mediators in the GATE cohort. AEBQ eating behaviours (emotional overeating, emotional undereating, hunger) partially mediated the association between BMI-GRS and BMI in the ALSPAC cohort. Sensitivity analyses revealed that lowering the reliability of eating behaviour resulted in variation of the proportion mediated by -1% to 10%. Additionally, depression score was an intermediate mediator in the GATE cohort (Supplementary Table S4, available as Supplementary data at *IJE* online).

**Table 1.** Mediation of body mass index genetic risk score and body mass index by eating behaviour^a^

	**a (Std.β, 95% CI)** ^b^		**b (Std.β, 95% CI)** ^c^		**c’ (direct effect of BMI-GRS on BMI, Std.β, 95% CI)** ^d^		**Indirect effect of BMI-GRS on BMI (Std.β, 95% CI)** ^e^		Proportion mediated (indirect/total effect)	
Eating behaviour	GATE	ALSPAC	GATE	ALSPAC	GATE	ALSPAC	GATE	ALSPAC	GATE	ALSPAC
**TFEQ-51** ^f^										
Disinhibition^g^	0.10 (0.05 to 0.14)	0.15 (0.10 to 0.20)	0.43 (0.37 to 0.48)	0.38 (0.34 to 0.43)	0.16 (0.14 to 0.19)		0.05 (0.03 to 0.07)		23%	
* Habitual disinhibition^g^*	0.07 (0.02 to 0.12)	0.10 (0.05 to 0.14)	0.39 (0.33 to 0.44)	0.26 (0.21 to 0.31)	0.18 (0.15 to 0.21)		0.04 (0.02 to 0.06)		18%	
* Emotional disinhibition^g^*	0.07 (0.02 to 0.12)	0.12 (0.08 to 0.17)	0.30 (0.25 to 0.35)	0.38 (0.33 to 0.42)	0.19 (0.15 to 0.23)		0.03 (0.01 to 0.04)		14%	
* Situational disinhibition^g^*	0.08 (0.03 to 0.13)	0.12 (0.07 to 0.17)	0.22 (0.17 to 0.27)	0.22 (0.17 to 0.27)	0.20 (0.17 to 0.22)		0.03 (0.01 to 0.04)		14%	
Hunger^h^	0.11 (0.06 to 0.16)	N/A	0.17 (0.12 to 0.23)	N/A	0.18 (0.13 to 0.22)	N/A	0.02 (0.01 to 0.03)	N/A	10%	N/A
* External hunger^h^*	0.11 (0.06 to 0.16)	N/A	0.17 (0.12 to 0.22)	N/A	0.18 (0.13 to 0.23)	N/A	0.02 (0.01 to 0.03)	N/A	10%	N/A
* Internal hunger^h^*	0.09 (0.04 to 0.14)	N/A	0.14 (0.08 to 0.19)	N/A	0.18 (0.14 to 0.23)	N/A	0.01 (0.001 to 0.02)	N/A	5%	N/A
**AEBQ**										
Emotional overeating^i^	N/A	0.09 (0.04 to 0.14)	N/A	0.26 (0.21 to 0.31)	N/A	0.21 (0.17 to 0.26)	N/A	0.02 (0.01 to 0.03)	N/A	9%
Emotional undereating^i^	N/A	–0.06 (–0.11 to –0.01)	N/A	–0.19 (–0.24 to –0.14)	N/A	0.22 (0.18 to 0.27)	N/A	0.01 (0.001 to 0.02)	N/A	4%
Enjoyment of food^i^	N/A	–0.03 (–0.08 to 0.02)	N/A	0.07 (0.02 to 0.12)	N/A	0.24 (0.19 to 0.28)	N/A	–0.002 (–0.01 to 0.002)	N/A	N/A^j^
Food fussiness^i^	N/A	0.02 (–0.03 to 0.07)	N/A	0.10 (0.05 to 0.15)	N/A	0.23 (0.18 to 0.28)	N/A	0.002 (–0.003 to 0.01)	N/A	0%
Food responsiveness^i^	N/A	0.01 (–0.04 to 0.06)	N/A	0.08 (0.03 to 0.13)	N/A	0.23 (0.19 to 0.28)	N/A	0.0002 (–0.004 to 0.004)	N/A	0%
Hunger^i^	N/A	–0.07 (–0.12 to –0.02)	N/A	–0.09 (–0.14 to –0.04)	N/A	0.23 (0.18 to 0.27)	N/A	0.01 (0.002 to 0.01)	N/A	4%
Satiety responsiveness^i^	N/A	–0.02 (–0.07 to 0.03)	N/A	–0.12 (–0.17 to –0.07)	N/A	0.23 (0.18 to 0.28)	N/A	0.002 (–0.004 to 0.01)	N/A	0%
Slowness in eating^i^	N/A	–0.03 (–0.08 to 0.02)	N/A	–0.12 (–0.16 to –0.07)	N/A	0.23 (0.18 to 0.28)	N/A	0.004 (–0.002 to 0.01)	N/A	0%

AEBQ, Adult Eating Behaviour Questionnaire; ALSPAC, Avon Longitudinal Study of Parents and Children study; Std.β, standardised beta coefficient; BMI, body mass index; BMI-GRS, body mass index genetic risk score; GATE, Genetics of Appetite study; TFEQ-51, Three-factor Eating Questionnaire-51 item; 95% CI, 95% confidence interval; N/A, not available.

a Structural equation model used to explore the association between BMI-GRS on eating behaviour, eating behaviour on BMI, and BMI-GRS std on BMI simultaneously (bootstrap 1000). Smoking, ethnicity, depression, anxiety, sex, age were controlled for in relationship a; ethnicity, depression, anxiety, sex and age controlled for in relationship b (Supplementary Figure S1, available as Supplementary data at *IJE* online). Model 1 (adjusted for sex and age only) results are available in Supplementary Table S3 (available as Supplementary data at *IJE* online).

b a represents the association between BMI-GRS and eating behaviour.

c b represents the association between the eating behaviour and BMI, adjusted for BMI-GRS.

d The direct effect (c’) is exposure of BMI-GRS on BMI while adjusting for the eating behaviour (the mediator). The indirect (or the mediation) effect is the product of a and b (a * b).

e The indirect effect quantifies how much of the effect of the BMI-GRS std on BMI goes through, or is mediated by, the eating behaviour.

f Italicized eating behaviours represent TFEQ-51 item subscales. The total effect is the sum of the direct and indirect effects of BMI-GRS std. The total effects were as follows.

g GATE and ALSPAC meta-mediation std. beta 0.22 (95% CI, 0.18 to 0.26), *n*=2687.

h TFEQ hunger items (GATE only) std. beta 0.20 (95% CI, 0.15 to 0.24), *n*=1219.

i AEBQ items (ALSPAC only) std. beta 0.23 (95% CI, 0.19 to 0.28), *n*=1468.

j Proportion mediated not shown due to inconsistent mediation (i.e. whereby the total effect is the sum of the (counteracting) direct and indirect effect, but the proportion mediated is a minus percentage and therefore illogical).

### Cognitive restraint as a moderator of the mediating effect

**Figure 3.**
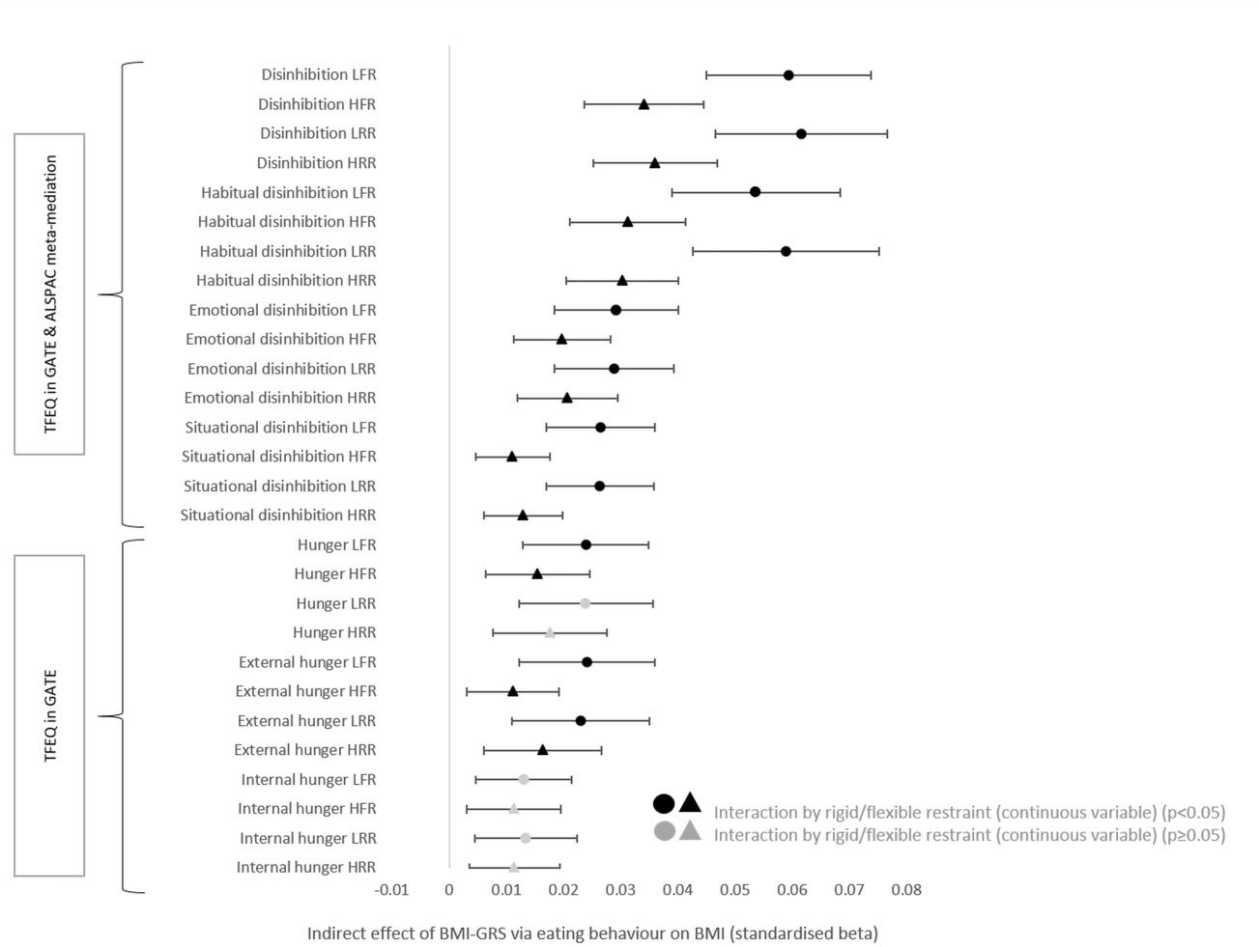
Indirect effect of body mass index genetic risk score mediated by TFEQ-51 eating behaviour (standardized), stratified by high and low flexible and rigid restraint. ALSPAC, Avon Longitudinal Study of Parents and Children; BMI, body mass index; BMI-GRS, body mass index genetic risk score; GATE, Genetics of Appetite study; TFEQ, Three-Factor Eating Questionnaire-51; LRR, low rigid restraint [restraint score categorized ‘low’ (=<3)]; HRR, high rigid restraint [restraint score categorized high (>3)]; LFR, low flexible restraint [restraint score categorized ‘low’ (=<3)]; HFR, high flexible restraint [restraint score categorized high (>3)]. The indirect effect was the product of a and b (a * b) which represents the mediation effect of the eating behaviour traits on the association between BMI-GRS and BMI. Black colour indicates flexible/rigid restraint interaction (as a continuous variable, *P* <0.05). Interactions were stratified into ‘high’ and ‘low’ flexible restraint (a score of ≤3 was low restraint, >3 was high restraint) to illustrate the effects of flexible restraint on the mediation in a binary fashion. Age and sex were controlled within each endogenous (dependent) variable in the structural model

**Figure 4.**
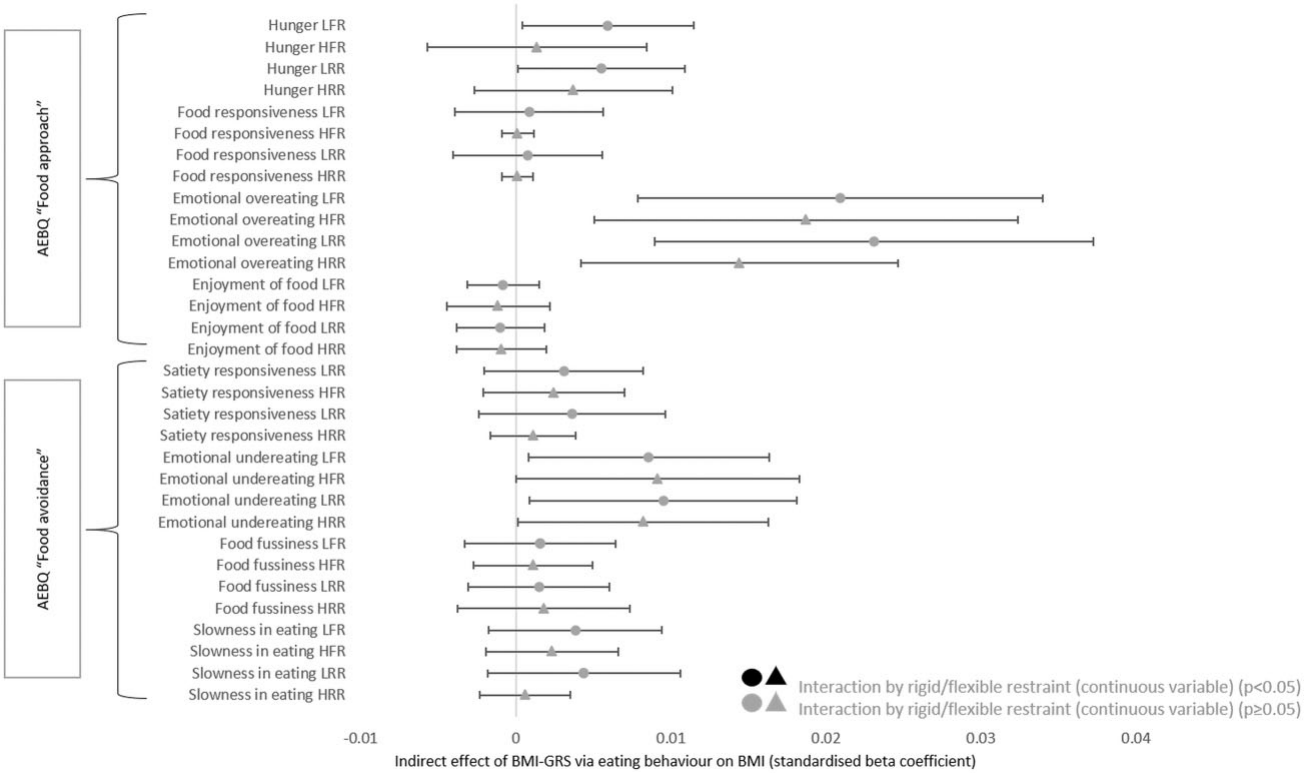
Indirect effect of body mass index genetic risk score mediated by AEBQ eating behaviour (standardized), stratified by high and low flexible and rigid restraint. AEBQ, Adult Eating Behaviour Questionnaire; ALSPAC, Avon Longitudinal Study of Parents and Children; BMI, body mass index; BMI-GRS, body mass index genetic risk score; GATE, Genetics of Appetite study; LRR, low rigid restraint [restraint score categorized ‘low’ (=<3]); HRR, high rigid restraint [restraint score categorized high (>3)]; LFR, low flexible restraint [restraint score categorized ‘low’ (=<3)]; HFR, high flexible restraint [restraint score categorized high (>3)]. The indirect effect was the product of a and b (a * b) which represents the mediation effect of the eating behaviour traits on the association between BMI-GRS and BMI. Black colour indicates flexible/rigid restraint interaction (as a continuous variable, *P* <0.05). Interactions were stratified into ‘high’ and ‘low’ flexible restraint (a score of ≤3 was low restraint, >3 was high restraint) to illustrate the effects of flexible restraint on the mediation in a binary fashion. Age and sex were controlled within each endogenous (dependent) variable in the structural model

Interaction analyses revealed meta-analysed GATE and ALSPAC rigid or flexible restraint did not directly moderate the association between BMI-GRS and BMI [rigid restraint: simple slope of BMI-GRS standardized beta (Std.β) 0.20, 95% confidence interval (CI) 0.16–0.24, simple slope of rigid restraint Std.β 0.07, 95% CI 0.02–0.11 and interaction Std.β 0.01, 95% CI -0.02–0.03/flexible restraint: simple slope of BMI-GRS Std.β 0.27, 95% CI 0.21 to 0.32, simple slope of flexible restraint Std.β -0.04, 95% CI -0.08 to -0.002 and interaction Std.β -0.02, 95% CI -0.04 to 0.002, adjusted for sex and age). However, moderation-mediation analyses revealed that both rigid and flexible restraint attenuated the association between some TFEQ-51 eating behaviours and BMI, i.e. pathway ‘b’ (habitual, emotional and situation disinhibition in the GATE and ALSPAC meta-mediated cohort, and external hunger in the GATE cohort, Figure 3, Figure 4). Mediation effects stratified by high versus low restraint are available in Tables 2 and 3.

**Table 2.** Mediation of body mass index genetic risk score and body mass index, stratified by flexible restraint^a^

Eating behaviour	**Total effect of BMI-GRS on BMI (Std.β, 95% CI)** ^b^		**Direct effect of BMI-GRS on BMI (Std.β, 95% CI)** ^c^		**Indirect effect (via eating behaviour) of BMI-GRS on BMI (Std.β, 95% CI)** ^d^				Interaction *P*
	LFR	HFR	LFR	HFR	LFR	% mediated	HFR	% mediated	
**TFEQ-51^e^**									
Disinhibition^f^	0.23 (0.18 to 0.27)	0.2 (0.16 to 0.24)	0.17 (0.12 to 0.21)	0.17 (0.12 to 0.21)	0.06 (0.04 to 0.07)	26	0.03 (0.02 to 0.04)	15	<0.01
*Habitual disinhibition*^f^	0.23 (0.18 to 0.28)	0.2 (0.16 to 0.25)	0.17 (0.13 to 0.22)	0.17 (0.13 to 0.22)	0.05 (0.04 to 0.07)	22	0.03 (0.02 to 0.04)	15	<0.01
*Emotional disinhibition*^f^	0.22 (0.17 to 0.27)	0.21 (0.16 to 0.27)	0.19 (0.14 to 0.25)	0.19 (0.14 to 0.25)	0.03 (0.02 to 0.04)	14	0.02 (0.01 to 0.03)	10	<0.01
*Situational disinhibition*^f^	0.22 (0.18 to 0.27)	0.21 (0.16 to 0.25)	0.2 (0.15 to 0.24)	0.2 (0.15 to 0.24)	0.03 (0.02 to 0.04)	14	0.01 (0 to 0.02)	5	<0.01
Hunger^g^	0.20 (0.16 to 0.24)	0.19 (0.15 to 0.23)	0.17 (0.13 to 0.21)	0.17 (0.13 to 0.21)	0.02 (0.01 to 0.03)	12	0.02 (0.01 to 0.02)	8	0.02
*External hunger*^g^	0.20 (0.16 to 0.24)	0.19 (0.15 to 0.23)	0.17 (0.14 to 0.21)	0.17 (0.14 to 0.21)	0.02 (0.01 to 0.04)	10	0.01 (0.003 to 0.02)	5	<0.01
*Internal hunger*^g^	0.20 (0.16 to 0.24)	0.19 (0.15 to 0.24)	0.18 (0.14 to 0.22)	0.18 (0.14 to 0.22)	0.01 (0.005 to 0.02)	5	0.01 (0.003 to 0.02)	5	0.317
**AEBQ**									
Emotional overeating^h^	0.25 (0.20 to 0.29)	0.25 (0.20 to 0.29)	0.23 (0.19 to 0.27)	0.23 (0.19 to 0.27)	0.02 (0.01 to 0.03)	8	0.02 (0.01 to 0.03)	8	0.37
Emotional undereating^h^	0.25 (0.20 to 0.29)	0.25 (0.20 to 0.29)	0.24 (0.20 to 0.28)	0.24 (0.20 to 0.28)	0.01 (0.0008 to 0.02)	4	0.01 (–0.00001 to 0.02)	0	0.87
Enjoyment of food^h^	0.25 (0.21 to 0.29)	0.25 (0.21 to 0.29)	0.25 (0.21 to 0.29)	0.25 (0.21 to 0.29)	–0.0008 (–0.003 to 0.001)	0	–0.001 (–0.004 to 0.002)	0	0.21
Food fussiness^h^	0.25 (0.21 to 0.29)	0.25 (0.21 to 0.29)	0.25 (0.21 to 0.29)	0.25 (0.21 to 0.29)	0.002 (–0.003 to 0.01)	0	0.001 (–0.003 to 0.01)	0	0.57
Food responsiveness^h^	0.25 (0.20 to 0.29)	0.25 (0.20 to 0.29)	0.25 (0.20 to 0.29)	0.25 (0.20 to 0.29)	0.0009 (–0.004 to 0.01)	0	0.0001 (–0.0009 to 0.001)	0	0.61
Hunger^h^	0.25 (0.21 to 0.29)	0.25 (0.20 to 0.29)	0.24 (0.20 to 0.28)	0.24 (0.20 to 0.28)	0.01 (0.0004 to 0.01)	4	0.001 (–0.01 to 0.01)	0	0.71
Satiety responsiveness^h^	0.25 (0.20 to 0.29)	0.25 (0.20 to 0.29)	0.24 (0.20 to 0.29)	0.25 (0.20 to 0.29)	0.003 (–0.002 to 0.01)	0	0.002 (–0.002 to 0.01)	0	0.82
Slowness in eating^h^	0.25 (0.21 to 0.29)	0.25 (0.20 to 0.29)	0.24 (0.20 to 0.28)	0.24 (0.20 to 0.28)	0.004 (–0.002 to 0.01)	0	0.002 (–0.002 to 0.01)	0	0.91

AEBQ, Adult Eating Behaviour Questionnaire; ALSPAC, Avon Longitudinal Study of Parents and Children; Std.β, standardized beta coefficient; BMI, body mass index; BMI-GRS, body mass index genetic risk score; GATE, Genetics of Appetite; HFR, high flexible restraint; LFR, low flexible restraint; TFEQ-51, Three-factor Eating Questionnaire-51; 95% CI, 95% confidence interval.

a Restraint was tested using a continuous interaction variable within the mediation SEM (e.g. flexible restraint*mediating eating behaviour) (bootstrap 1000) (Figures 3 and 4). Mediation models shown here were stratified into ‘high’ and ‘low’ flexible restraint (a score of ≤3 was low restraint, >3 was high restraint (Aurelie *et al.*, 2012; Kruger *et al*., 2016) to illustrate the effects of flexible restraint on the mediation in a binary fashion. Models were adjusted for age and sex. Pathways a and b (standardized beta coefficient, 95% confidence interval) are available in Supplementary Table S5 (available as Supplementary data at *IJE* online).

b The total effect is the sum of the direct and indirect effects of BMI-GRS std.

c The direct effect (c’) is exposure of BMI-GRS std on BMI while adjusting for the eating behaviour (the mediator).

d The indirect (or the mediation) effect is the product of a and b (a * b). In the indirect effect quantifies how much of the effect of the BMI-GRS std on BMI goes through, or is mediated by, the eating behaviour.

e Italicized eating behaviours represent TFEQ-51 item subscales.

f GATE and ALSPAC meta-mediation LFR *n*=2661, HFR *n*=1119.

g LFR *n*=1380, HFR *n*=721.

h LFR *n*=1281, HFR *n*=398.

**Table 3.** Mediation of body mass index genetic risk score and body mass index, stratified by rigid restraint^a^

Eating behaviour	**Total effect of BMI-GRS on BMI (Std.β, 95% CI)** ^b^		**Direct effect of BMI-GRS on BMI (Std.β, 95% CI)** ^c^		**Indirect effect (via eating behaviour) of BMI-GRS on BMI (Std.β, 95% CI)** ^d^				Interaction *P*
	LRR	HRR	LRR	HRR	LRR	% mediated	HRR	% mediated	
**TFEQ-51^e^**									
Disinhibition^f^	0.23 (0.17 to 0.29)	0.2 (0.16 to 0.25)	0.17 (0.11 to 0.22)	0.17 (0.11 to 0.22)	0.06 (0.05 to 0.08)	26	0.04 (0.03 to 0.05)	20	<0.01
*Habitual disinhibition*^f^	0.23 (0.17 to 0.29)	0.2 (0.15 to 0.25)	0.17 (0.12 to 0.23)	0.17 (0.12 to 0.23)	0.06 (0.04 to 0.08)	26	0.03 (0.02 to 0.04)	15	<0.01
*Emotional disinhibition*^f^	0.22 (0.17 to 0.28)	0.22 (0.17 to 0.26)	0.19 (0.14 to 0.25)	0.19 (0.14 to 0.25)	0.03 (0.02 to 0.04)	14	0.02 (0.01 to 0.03)	9	<0.01
*Situational disinhibition*^f^	0.22 (0.17 to 0.28)	0.21 (0.16 to 0.26)	0.20 (0.15 to 0.25)	0.20 (0.15 to 0.25)	0.03 (0.02 to 0.04)	14	0.01 (0.01 to 0.02)	5	<0.01
Hunger^g^	0.20 (0.16 to 0.24)	0.19 (0.15 to 0.23)	0.17 (0.13 to 0.21)	0.17 (0.13 to 0.21)	0.02 (0.01 to 0.03)	10	0.02 (0.01 to 0.03)	11	0.11
*External hunger*^g^	0.20 (0.16 to 0.23)	0.19 (0.15 to 0.23)	0.17 (0.13 to 0.21)	0.17 (0.13 to 0.21)	0.02 (0.01 to 0.03)	12	0.02 (0.01 to 0.03)	9	0.03
*Internal hunger*^g^	0.19 (0.16 to 0.23)	0.19 (0.15 to 0.23)	0.18 (0.14 to 0.22)	0.18 (0.14 to 0.22)	0.01 (0.005 to 0.02)	5	0.01 (0.004 to 0.02)	5	0.43
**AEBQ**									
Emotional overeating^h^	0.25 (0.21 to 0.29)	0.24 (0.20 to 0.28)	0.23 (0.19 to 0.27)	0.23 (0.19 to 0.27)	0.02 (0.01 to 0.04)	4	0.01 (0.004 to 0.02)	4	0.32
Emotional undereating^h^	0.25 (0.21 to 0.29)	0.25 (0.20 to 0.29)	0.24 (0.20 to 0.28)	0.24 (0.20 to 0.28)	0.01 (0.0009 to 0.02)	4	0.01 (0.0001 to 0.02)	4	0.95
Enjoyment of food^h^	0.25 (0.21 to 0.29)	0.25 (0.21 to 0.29)	0.25 (0.21 to 0.29)	0.25 (0.21 to 0.29)	–0.001 (–0.004 to 0.002)	0	–0.001 (–0.004 to 0.002)	0	0.97
Food fussiness^h^	0.25 (0.2 to 0.29)	0.25 (0.21 to 0.29)	0.25 (0.20 to 0.29)	0.25 (0.20 to 0.29)	0.001 (–0.003 to 0.01)	0	0.002 (–0.004 to 0.01)	0	0.94
Food responsiveness^h^	0.25 (0.21 to 0.29)	0.25 (0.21 to 0.29)	0.25 (0.21 to 0.29)	0.25 (0.21 to 0.29)	0.0008 (–0.004 to 0.01)	0	0.0001 (–0.0009 to 0.001)	0	0.14
Hunger^h^	0.25 (0.21 to 0.29)	0.25 (0.20 to 0.29)	0.24 (0.20 to 0.28)	0.24 (0.20 to 0.28)	0.01 (0.0001 to 0.01)	4	0.004 (–0.003 to 0.01)	0	0.53
Satiety responsiveness^h^	0.25 (0.21 to 0.29)	0.25 (0.21 to 0.29)	0.25 (0.21 to 0.29)	0.25 (0.21 to 0.29)	0.004 (–0.002 to 0.01)	0	0.001 (–0.002 to 0.01)	0	0.14
Slowness in eating^h^	0.25 (0.21 to 0.29)	0.24 (0.2 to 0.29)	0.24 (0.20 to 0.29)	0.24 (0.20 to 0.29)	0.004 (–0.002 to 0.01)	0	0.0006 (–0.002 to 0.004)	0	N/A^i^

AEBQ, Adult Eating Behaviour Questionnaire; ALSPAC, Avon Longitudinal Study of Parents and Children; Std.β, standardized beta coefficient; BMI, body mass index; BMI-GRS, body mass index genetic risk score; GATE, Genetics of Appetite; HRR, high rigid restraint; LRR, low rigid restraint; TFEQ-51, Three-factor Eating Questionnaire-51; 95% CI, 95% confidence interval.

a Restraint was tested using a continuous interaction variable within the mediation SEM (e.g. rigid restraint*mediating eating behaviour) (bootstrap 1000) (Figures 3 and 4). Mediation models shown here were stratified into ‘high’ and ‘low’ rigid restraint [a score of ≤3 was low restraint, >3 was high restraint (Aurelie *et al.,* 2012; Kruger *et al.*, 2016] to illustrate the effects of rigid restraint on the mediation in a binary fashion. Models were adjusted for age and sex. Pathways a and b (standardized beta coefficient, 95% confidence interval) are available in Supplementary Table S5 (available as Supplementary data at *IJE* online).

b The total effect is the sum of the direct and indirect effects of BMI-GRS std.

c The direct effect (c’) is exposure of BMI-GRS std on BMI while adjusting for the eating behaviour (the mediator).

d The indirect (or the mediation) effect is the product of a and b (a * b). In the indirect effect quantifies how much of the effect of the BMI-GRS std on BMI goes through, or is mediated by, the eating behaviour.

e Italicized eating behaviours represent TFEQ-51 item subscales.

f GATE and ALSPAC meta-mediation LRR *n*=2661, HRR *n*=1119.

g LRR *n*=1418, HRR *n*=683.

h LRR *n*=1243, HRR *n*=436.

i The interaction *P-*value was 0.05, indicating a small difference in the indirect effect between participants in the ‘low’ versus ‘high’ rigid restraint groups. However, as the indirect effect indicates, slowness in eating is not a partial mediator of the relationship between BMI-GRS and BMI in in both groups (i.e. coefficient confidence intervals include 0), we do not report rigid restraint as interacting with the influence of slowness in eating.

The GATE and ALSPAC study samples (participants with data for eating behaviour, BMI and BMI-GRS) consisted of 2101 (61% females) and 1679 (68% females) participants, respectively (Table 4). Participants included in Model 1 of the GATE study were younger and lower BMI compared with those with who were excluded due to missing data (*P *<0.05). Participants included in Model 1 of the ALSPAC study were more likely to be female compared with participants in the original ALSPAC birth cohort with all BMI-genetic risk score data (*P *<0.05). Missingness of ALSPAC participants with TFEQ data at age 25 years was associated with lower BMI at 24 (*P *<0.05).

**Table 4.** Participant characteristics and eating behaviour traits in the Genetics of Appetite (GATE) and Avon Longitudinal Study of Parents and Children (ALSPAC) studies

Characteristic	**GATE (baseline visit)** ^a^		**GATE (online questionnaire)** ^b^		**ALSPAC at 23** ^c^		ALSPAC at 25^d^	
	Mean (SD)/median (IQR)/*n* (%)	***n*** ^e^	Mean (SD)/median (IQR)/*n* (%)	***n*** ^e^	Mean (SD)/median (IQR)/*n* (%)	***n*** ^f^	Mean (SD)/median (IQR)/*n* (%)	***n*** ^f^
Age (years)^g^	59.00 (47.00, 66.00)	2101	62.00 (51.00, 69.00)	2101	23.83 (23.42, 24.25)	1679		
Currently smoking^h^	50 (4.10)	1219	N/A		181 (12.33)	1468	N/A	
Ethnic origin		1219	N/A			1468	N/A	
White	1211 (99.34)				1465 (99.80)			
Non-White	8 (0.66)				<5 (0.20)			
BMI^g^^,^^h^	25.44 (22.98, 28.62)	2101	N/A		23.42 (21.33, 26.68)	1679	N/A	
Obese (BMI ≥30 kg/m^2^)	380 (18.09)	2101	N/A		208 (12.39)	1679	N/A	
BMI-GRS std^i^	–0.00 (1.00)	2102	N/A		–0.06 (1.01)	1679	N/A	
**AEBQ**								
Emotional overeating	N/A		N/A		2.64 (0.96)	1679	N/A	
Emotional undereating	N/A		N/A		2.80 (0.98)	1679	N/A	
Enjoyment of food	N/A		N/A		4.44 (0.69)	1679	N/A	
Food fussiness	N/A		N/A		2.06 (0.92)	1679	N/A	
Food responsiveness	N/A		N/A		3.35 (0.79)	1679	N/A	
Hunger	N/A		N/A		3.05 (0.75)	1679	N/A	
Satiety responsiveness	N/A		N/A		2.39 (0.83)	1679	N/A	
Slowness in eating	N/A		N/A		2.39 (0.85)	1679	N/A	
**TFEQ-51** ^j^								
Cognitive restraint	N/A		9.12 (4.60)	2101	N/A		N/A	
*Flexible cognitive restraint*	N/A		2.91 (1.73)	2101	N/A		2.30 (1.80)	1679
*Rigid cognitive restraint*	N/A		2.76 (1.87)	2101	N/A		2.39 (1.87)	1679
Disinhibition	N/A		5.65 (3.89)	2101	N/A		6.75 (3.88)	1679
*Habitual disinhibition*	N/A		0.96 (1.36)	2101	N/A		1.05 (1.41)	1679
*Emotional disinhibition*	N/A		1.07 (1.22)	2101	N/A		1.17 (1.22)	1679
*Situational disinhibition*	N/A		2.11 (1.52)	2101	N/A		2.65 (1.54)	1679
Hunger	N/A		4.20 (3.23)	2101	N/A		N/A	
*External hunger*	N/A		1.71 (1.56)	2101	N/A		N/A	
*Internal hunger*	N/A		1.50 (1.71)	2101	N/A		N/A	

AEBQ, Adult Eating Behaviour Questionnaire; ALSPAC, Avon Longitudinal Study of Parents and Children; BMI, body mass index; BMI-GRS std, body mass index genetic risk score (standardized); GATE, Genetics of Appetite; IQR, interquartile range; SD, standard deviation; TFEQ-51, Three-Factor Eating Questionnaire; N/A, not available.

a GATE study: collected by clinical research team during the baseline visit required to partake in the wider EXETER 10 000 study. Analyses sample consists of participants with available data for BMI, all TFEQ-51 items and DNA data.

b GATE study: self-reported questionnaire. A number of participants answered the questionnaire twice (due to additional recruitment); responses from their first questionnaire were used. Analysis sample consisted of participants with available data for BMI, TFEQ-51 and DNA data.

c ALSPAC study: analysis sample consisted of participants with available data for all AEBQ items (collected during the self-reported ‘Me at 23+ questionnaire’), BMI at 24 years and DNA data.

d ALSPAC: analysis sample consisted of participants with available data for TFEQ-51 items: habitual, situational, emotional disinhibition, rigid restraint and flexible restraint (collected during the self-reported ‘Life at 25+’’), BMI at 24 years and DNA data.

e GATE: *n* for confounders included in Model 2 may differ from *n* in Model 1 (Model 1 *n*=2101, Model 2 *n*=1219) due to missing data for confounders.

f ALSPAC: *n* for confounders included in Model 2 may differ from *n* in Model 1 (Model 1 *n*=1679, Model 2 *n*=1468) due to missing data for confounders.

g Median (25th percentile to 75th percentile).

h ALSPAC: measured during ‘Focus 24+’ Focus Clinic.

i BMI-GRS was quantified by calculating the sum of the total number of risk alleles at 605 (GATE) and 934 (ALSPAC) of the 941 BMI-associated loci reported in recent GWAS studies, weighted by their estimated effect sizes (Locke *et al*., 2015; Yengo *et al*., 2018). Standardized BMI-genetic risk scores were calculated.

j Questions available in italicized items represent subscales created from original TFEQ-51 questionnaire.

## Discussion

### Summary of findings

Our study findings support our hypothesis that eating behaviours are a key behavioural pathway in which risk variants associated with obesity influence BMI. Furthermore, restrictive eating may play an important role in offsetting high genetic risk to obesity.

### Implications of findings

We showed BMI-GRS and BMI associations were partially mediated by disinhibition and hunger. Compared with recent studies, we reported smaller indirect estimates (proportion mediated by disinhibition 23% versus 36% and hunger 10% versus 16%, in the present study compared with Jacob *et al*.,^18^ respectively). Contrary to the current study, Jacob *et al*.^18^ did not find that emotional disinhibition was a mediator of the relationship between genetic risk for obesity and BMI (14% in the present study versus 0% in the Jacob study). However, mediation of BMI-GRS and BMI through emotional eating (similar to emotional disinhibition in this study) was shown in four other cohorts (10–13% mediated).^15^^,^^18^^,^^42^ Our findings show the mediation of BMI-GRS on BMI was most strongly mediated by habitual disinhibition (18%). This may be the most frequent pathway in which BMI-genetic mechanisms are realized, because it measures tendency to overeat in response to daily life circumstances and there are a high level of daily overeating opportunities within the Western obesogenic environment.^20^

Furthermore, this study revealed emotional eating (i.e. under- and over-eating) and hunger were mediators. To our knowledge, one thesis publication used AEBQ in adults, reporting emotional over-/undereating and slowness in eating were mediators for the genetic risk to obesity.^16^ In contrast, earlier studies using the Child Eating Behaviour questionnaire found that satiety responsiveness, food responsiveness and enjoyment of food were mediators of the genetic risk to obesity and adiposity.^7^^,^^17^^,^^43^ Our findings suggest that the role of satiety responsiveness and other behaviours reported in childhood pathways may not persist into adulthood.^16^ Instead, eating behaviours such as uncontrolled eating and hunger are likely to become more prominent.

Restraint as a moderator has been investigated before.^15^^,^^20^ Flexible restraint attenuated the influence of habitual disinhibition on BMI more than emotional and situational disinhibition.^20^ However, a key strength and novelty of our study was to detail the positive moderating impact of rigid restraint, as well flexible restraint, on the effects of high genetic risk to obesity in a wide age group (22 to 92 years old), well-validated eating behaviour measures and recently published variants to calculate genetic risk for obesity.^6^ Notably, we did not find that the either restraint type moderated the direct pathway between BMI-GRS and BMI; instead, we discovered both rigid and flexible restraint moderated the indirect pathway mediated via disinhibited eating and external hunger. Our results suggest that restraint (whether it be rigid or flexible) may ‘counter’ some of the predisposition to BMI via disinhibition and external hunger. Whereasthis association was expected for flexible restraint, which is regarded as ‘adaptive’ and was negatively associated with BMI here, it was not anticipated for rigid restraint, which is often seen as unhelpful and was positively associated with BMI.^20^^,^^21^^,^^44^

Consistent with the direction of the models tested in this study, previous studies have shown that disinhibition and susceptibility to hunger were predictors of weight gain in a longitudinal study and restraint as an adaptive tool to the consequence of weight gain.^20^^,^^45^ However, the relationship between eating behaviour and obesity is known to be complex and bidirectional. The influence of genetic risk can only be one-sided but the expression of genes can be altered by environmental factors and aging. We included well-known confounders used in earlier studies to reduce bias from potential omitted confounders. These did not eliminate but did reduce the indirect pathway between BMI-GRS and BMI. A similar study including socioeconomic status reported findings similar to our analyses (e.g. indirect mediation proportion: emotional overeating 8% versus 9% and emotional undereating 3% versus 4%) indicating pathway b is unlikely to be overestimated in the present study.^16^ Inter-individual variability was tested in a subsample of the present study. Eating behaviour scores remained stable between two time points, indicating high test-retest reliability. However, sensitivity analyses of eating behaviour demonstrated that with less than perfect reliability, some associations are likely downwardly biased (i.e. pathway b is likely underestimated and pathway c’ is likely overestimated). Given the large sample size of the present cohorts, direct observations were not possible. Further research is required to specify omitted confounders, in addition to increasing reliability and accuracy of eating behaviour measurements.

The ability to identify high-risk individuals may facilitate targeted obesity-prevention interventions. However, a challenge to BMI-genetic risk scores is that they account for a lower proportion of phenotypic variability compared with BMI heritability estimates. Recent studies have found an increasing number of loci associated with eating behaviours which account for a higher proportion of the variance in BMI (e.g. Abdulkadir 2020^46^), yet these are not close to heritability estimates and cannot rule out other genetic sources of obesity e.g. taste and real food intake^47^ (these were of scope or not available to test in this study). Sensitivity analyses also revealed that depression was an intermediate mediator in the GATE cohort, which indicates some BMI-risk genes are pleiotropic,^48^ and GRS amalgamates all genetically influenced functions contributing to BMI. The inclusion of depression score in the BMI-GRS-eating behaviour-BMI model was necessary as an established confounder related to both eating behaviour and BMI,^49^ but this may have introduced bias to the indirect effect by blocking some of the effect of BMI-GRS on BMI mediated via eating behaviours. Further research is required to understand the full extent of the overlap, the interaction between outcomes and shared genetic factors. Additionally, the GATE study BMI-GRS (but not ALSPAC) was computed before the latest version of the GWAS for BMI was published, which identified a further ∼300 SNVs.^6^ Internal standardized BMI-GRS enabled comparison of BMI-GRS between cohorts, but we did not use external standardization, which limited the comparability to other studies. However, we only found an additional 0.8% increase in variation explained by ALSPAC BMI-GRS for BMI (ALSPAC r squared value 6.9% versus GATE 6.1%) using the latest GWAS.^6^ Moreover, previous studies have reported consistent associations between BMI-GRS, eating behaviours and BMI, despite using varying numbers of SNVs.^12^^,^^14–16^^,^^18^^,^^42^ Both study cohorts comprised a wide range of age and BMI, but representation was limited to overwhelmingly White participants in the South West of England. Although this may limit the generalizability of the results, we expect minimal genetic bias, as the within-region structure is considerably smaller and ancestry is more homogeneous than at the Europe-wide level reported in other studies. Finally, analyses were not adjusted for multiple testing due to correlated phenotypes, which may have increased the chances of false-positive findings considering the high number of tests conducted.

## Conclusion

In summary, eating behaviours such as disinhibited eating, internal/external hunger and emotional over-/undereating, mediate the genetic susceptibility to obesity by 4% to 23%. For the first time, we show that cognitive restriction of eating using flexible or rigid strategies reduced the indirect association between genetic risk to obesity and BMI mediated by eating behaviours across all adult age groups in two large UK cohorts. Subpopulations in which restrictive eating (flexible or rigid) is most helpful to overcome predisposition to obesity and uncontrolled eating should be further investigated in longitudinal studies. Furthermore, public health interventions could seek to identify strategies that help individuals cope with urges to overeat.

## Ethics approval

The study was conducted according to the Helsinki Declaration. Ethical approval for EXETER 10 000 and Genetics of Appetite Study was provided by National Research Ethics Service (NRES) South West Ethics Committee and the Peninsula Research Bank.^50^ Ethical and consent approval for the Avon Longitudinal Study of Parents and Children was obtained from the ALSPAC Ethics and Law Committee and the local research ethics committees. No individual-level consent was required, and all data were anonymized.

## Supplementary Material

dyad092_Supplementary_Data

## Data Availability

The data underlying this article are available upon request to the ALSPAC data portal. The ALSPAC data management plan describes in detail the policy regarding data sharing, which is through a system of managed open access. Full instructions for applying for data access can be found here: [http://www.bristol.ac.uk/alspac/researchers/access/].
