# Supplementary material for: Mediation and moderation of genetic risk of obesity through eating behaviours in two UK cohorts
Source: Int J Epidemiol. 2023 Jul 6;52(6):1926–38. doi: 10.1093/ije/dyad092 (PMC10749755; doi:10.1093/ije/dyad092)
Supplement: dyad092_Supplementary_Data [file dyad092_supplementary_data.docx]

# Online Supporting Material: supplementary information

Ethnicity^2^

Sex^1,2^

Age^1,2^

Sex^1,2^

Age^1,2^

Anxiety^2^

Anxiety^2^

Depression^2^

Depression^2^

Smoking^2^

Ethnicity^2^

BMI-GRS

BMI

Eating Behaviours

c’

a

b

Figure S1: the effect of BMI-genetic risk on BMI, mediated by eating behaviour (model 1 and model 2)

BMI-GRS, body-mass-index genetic risk score

^1^Model 1

^2^Model 2

a represents the linear regression association between BMI-GRS and eating behaviour, b represents the linear regression association between eating behaviour and BMI, c’ represents the association between BMI-GRS and BMI when eating behaviour is included in the model (the direct effect). The product of a and b (a * b) represents the mediation effect (indirect effect) of the eating behaviour traits on the association between BMI-GRS and BMI. Model 1: age and sex were controlled for with each endogenous (dependent) variable in the structural model. Model 2: smoking, ethnicity, depression, anxiety, sex, age were controlled for in relationship a; ethnicity, depression, anxiety, sex and age controlled for in relationship b.

BMI-GRS

BMI

Eating Behaviours

c’

a

b

Rigid restraint/

Flexible restraint * eating behaviour interaction

Age

Sex

Age

Sex

Rigid restraint/

Flexible restraint * eating behaviour interaction

Figure S2: the effect of BMI-genetic risk on BMI, mediated by eating behaviour and moderated by rigid or flexible restraint

BMI-GRS, body-mass-index genetic risk score

a represents the linear regression association between BMI-GRS and eating behaviour, b represents the linear regression association between eating behaviour and BMI, c’ represents the association between BMI-GRS and BMI when eating behaviour is included in the model (the direct effect). The product of a and b (a * b) represents the mediation effect (indirect effect) of the eating behaviour traits on the association between BMI-GRS and BMI. Age and sex were controlled for with each endogenous (dependent) variable in the structural model. Moderated of the ‘b’ pathway by restraint was tested using a continuous interaction variable.

AEBQ “Food avoidance” ALSPAC study

AEBQ “Food approach” ALSPAC study

TFEQ GATE study

TFEQ GATE & ALSPAC studies meta-mediation

Figure S3: indirect effect of BMI-GRS mediated by eating behaviour (standardised, model 1 adjusted for age and sex)

ALSPAC, Avon Longitudinal Study of Parents and Children; BMI, body-mass-index; BMI-GRS, body-mass-index genetic risk score; GATE, Genetics of Appetite study

Model 1: adjusted age and sex were controlled within each endogenous (dependent) variable in the structural model

|  | Number of items | Range of score^1^ | Cronbach’s alpha | |
| --- | --- | --- | --- | --- |
| TFEQ |  |  | GATE | ALSPAC |
| Flexible restraint^2^ | 7 | 0-7 | 0.6142 | 0.6970 |
| Rigid restraint ^2^ | 7 | 0-7 | 0.6846 | 0.7171 |
| Habitual disinhibition^2^ | 5 | 0-5 | 0.7640 | 0.7424 |
| Emotional disinhibition^2^ | 3 | 0-3 | 0.8153 | 0.7857 |
| Situational disinhibition^2^ | 5 | 0-4 | 0.6668 | 0.6388 |
| External hunger^2^ | 6 | 0-5 | 0.6366 | N/A |
| Internal hunger^2^ | 6 | 0-6 | 0.7655 | N/A |
| AEBQ |  |  |  |  |
| Emotional overeating | 5 | 0-5 | N/A | 0.8823 |
| Emotional undereating | 5 | 0-5 | N/A | 0.9000 |
| Enjoyment of food | 3 | 0-3 | N/A | 0.9006 |
| Food fussiness | 5 | 0-5 | N/A | 0.9136 |
| Food responsiveness | 4 | 0-4 | N/A | 0.7467 |
| Hunger | 5 | 0-5 | N/A | 0.6859 |
| Satiety responsiveness | 4 | 0-4 | N/A | 0.7994 |
| Slowness in eating | 4 | 0-4 | N/A | 0.7626 |

Table S1: eating behaviour questionnaire internal consistency (Cronbach's Alpha)

AEBQ, Adult Eating Behaviour Questionnaire; ALSPAC, Avon Longitudinal Study of Parents and Children; Genetics of Appetite, GATE; Three-factor Eating Questionnaire-52

^1^The maximum of the range is not sum of the total number of items in selected eating behaviour

^2^Within the GATE study, TFEQ-51 items were collected twice for 531 individuals who repeated the questionnaire during the second recruitment invitation, the first TFEQ-51 score was used as the mediator in the present study. The median time between the first completed TFEQ and second TFEQ was 3.83 years (IQR 3.78 to 3.90 years). TFEQ item scores were similar across both time points for repeat participants (correlation coefficient range 0.62 to 0.76, p<0.001 for all tests). Therefore, the first TFEQ-51 score was used as the mediator in the present study.

Table S2: additional variables included in the GATE and ALSPAC studies

| Variable | Description | |
| --- | --- | --- |
|  | GATE study | ALSPAC study |
| Smoking | Currently smoking: yes, no | Currently smoking: yes, no |
| Depression | Patient-Health Questionnaire-9: 0-4 none, 5-9 mild, 10-14 moderate, 15-19 moderately severe, 20-27 severe. | Modified version of the long Mood and Feelings Questionnaire. Me at 23+ included 18/33 items and Life at 25+ included 17/33 items. |
| Anxiety | Generalised Anxiety Disorder Assessment-7: 5, 10, and 15 are cut-off points for mild, moderate and severe anxiety, respectively | Generalised anxiety disorder: yes, no |
| Sex | Male, female | Male, female |
| Age | Years | Years |
| Ethnicity | White, non-white | White, non-white |

ALSPAC, Avon Longitudinal Study of Parents and Children; BIA, Bioelectrical impedance; GATE, Genetics of Appetite Study

Table S3: Mediation of BMI-GRS and BMI (model 1 adjusted for age and sex only)^1^

| Eating behaviour | a (Std.β, 95% CI)^2^ | | b (Std.β, 95% CI)^3^ | | c’ (direct effect of BMI-GRS on BMI, Std.β, 95% CI)^4^ | | Indirect effect of BMI-GRS on BMI (Std.β, 95% CI)^5^ | | Proportion mediated (indirect/total effect) | |
| --- | --- | --- | --- | --- | --- | --- | --- | --- | --- | --- |
| TFEQ-51_6_ | GATE | ALSPAC | GATE | ALSPAC | GATE | ALSPAC | GATE | ALSPAC | GATE | ALSPAC |
| Disinhibition^7^ | 0.11 (0.07 to 0.15) | 0.15 (0.10 to 0.19) | 0.47 (0.43 to 0.51) | 0.40 (0.36 to 0.45) | 0.17 (0.13 to 0.22) | | 0.05 (0.04 to 0.06) | | 23% | |
| *Habitual disinhibition*^7^ | 0.10 (0.06 to 0.14) | 0.13 (0.08 to 0.17) | 0.44 (0.41 to 0.48) | 0.40 (0.36 to 0.45) | 0.17 (0.13 to 0.22) | | 0.05 (0.03 to 0.06) | | 23% | |
| *Emotional disinhibition*^7^ | 0.08 (0.04 to 0.12) | 0.10 (0.05 to 0.14) | 0.37 (0.33 to 0.41) | 0.28 (0.24 to 0.33) | 0.20 (0.14 to 0.26) | | 0.03 (0.02 to 0.04) | | 14% | |
| *Situational disinhibition*^7^ | 0.07 (0.03 to 0.12) | 0.11 (0.06 to 0.16) | 0.29 (0.25 to 0.33) | 0.25 (0.20 to 0.29) | 0.20 (0.14 to 0.26) | | 0.02 (0.01 to 0.03) | | 9% | |
| Hunger^8^ | 0.10 (0.06 to 0.14) | N/A | 0.24 (0.20 to 0.28) | N/A | 0.17 (0.13 to 0.21) | N/A | 0.02 (0.01 to 0.03) | N/A | 10% | N/A |
| *External hunger*^8^ | 0.09 (0.05 to 0.13) | N/A | 0.24 (0.20 to 0.28) | N/A | 0.17 (0.14 to 0.21) | N/A | 0.02 (0.01 to 0.03) | N/A | 10% | N/A |
| *Internal hunger*^8^ | 0.08 (0.04 to 0.12) | N/A | 0.18 (0.14 to 0.23) | N/A | 0.18 (0.15 to 0.22) | N/A | 0.01 (0.01 to 0.02) | N/A | 5% | N/A |
| AEBQ |  |  |  |  |  |  |  |  |  |  |
| Emotional overeating^9^ | N/A | 0.08 (0.03 to 0.13) | N/A | 0.27 (0.23 to 0.32) | N/A | 0.23 (0.19 to 0.27) | N/A | 0.02 (0.008 to 0.04) | N/A | 8% |
| Emotional undereating^9^ | N/A | -0.05 (-0.10 to -0.003) | N/A | -0.20 (-0.24 to -0.15) | N/A | 0.24 (0.2 to 0.28) | N/A | 0.009 (0.0003 to 0.02) | N/A | 4% |
| Enjoyment of food^9^ | N/A | -0.02 (-0.06 to 0.03) | N/A | 0.05 (0.01 to 0.10) | N/A | 0.25 (0.21 to 0.29) | N/A | -0.001 (-0.004 to 0.002) | N/A | 0% |
| Food fussiness^9^ | N/A | 0.01 (-0.03 to 0.06) | N/A | 0.10 (0.06 to 0.15) | N/A | 0.25 (0.21 to 0.29) | N/A | 0.001 (-0.003 to 0.006) | N/A | 0% |
| Food responsiveness^9^ | N/A | 0.01 (-0.04 to 0.06) | N/A | 0.08 (0.04 to 0.13) | N/A | 0.25 (0.21 to 0.29) | N/A | 0.001 (-0.003 to 0.005) | N/A | 0% |
| Hunger^9^ | N/A | -0.06 (-0.11 to -0.02) | N/A | -0.09 (-0.14 to -0.05) | N/A | 0.25 (0.2 to 0.29) | N/A | 0.005 (0.00002 to 0.01) | N/A | 2% |
| Satiety responsiveness^9^ | N/A | -0.03 (-0.07 to 0.02) | N/A | -0.12 (-0.17 to -0.07) | N/A | 0.25 (0.21 to 0.29) | N/A | 0.003 (-0.002 to 0.008) | N/A | 0% |
| Slowness in eating^9^ | N/A | -0.03 (-0.08 to 0.01) | N/A | -0.12 (-0.16 to -0.07) | N/A | 0.25 (0.21 to 0.29) | N/A | 0.004 (-0.001 to 0.008) | N/A | 0% |

AEBQ, Adult Eating Behaviour Questionnaire; ALSPAC, Avon Longitudinal Study of Parents and Children; Std.β, standardised beta coefficient; BMI, body-mass-index; BMI-GRS, body-mass-index genetic risk score; GATE, Genetics of Appetite; TFEQ-51, Three-factor Eating Questionnaire-51 item; 95% CI, 95% confidence interval

^1^Structural equation model used to explore the association between BMI-GRS on eating behaviour, eating behaviour on BMI, and BMI-GRS std on BMI simultaneously (bootstrap 1,000).

^2^a represents the association between BMI-GRS and eating behaviour.

^3^b represents the association between the eating behaviour and BMI, adjusted for BMI-GRS. Sex and age were controlled for in relationship a and b (**Supplementary Figure 1**).

^4^The direct effect (c’) is exposure of BMI-GRS on BMI while adjusting for the eating behaviour (the mediator). The indirect (or the mediation) effect is the product of a and b (a * b).

^5^The indirect effect quantifies how much of the effect of the BMI-GRS std on BMI goes through, or is mediated by, the eating behaviour (see **Supplementary Figure 3** for illustration).

^6^Italicised eating behaviours represent TFEQ-51 item subscales

The total effect is the sum of the direct and indirect effects of BMI-GRS std. The total effect were as follows: ^7^GATE and ALSPAC meta-mediation std. beta 0.22 (95%CI 0.18 to 0.27), n=3780; ^8^TFEQ hunger items (GATE only) std. beta 0.20 (95%CI 0.16 to 0.23) n=2101, ^9^AEBQ items (ALSPAC only) std. beta 0.25 (95%CI 0.21 to 0.29), n=1679

Table S4: Mediation of BMI-GRS and BMI, sensitivity analyses

| **Measurement error for eating behaviour^1^** | a (Std.β)^2^ | b (Std.β)^3^ | Direct effect (Std.β)  c'^4^ | Indirect effect of BMI-GRS on BMI (Std.β)^5^ | Total effect (Std.β)  C^6^ | Proportion mediated (indirect/total effect)  c-c'/c |
| --- | --- | --- | --- | --- | --- | --- |
| **GATE** |  |  |  |  |  |  |
| Disinhibition^7^ | 0.10 | 0.59 | 0.14 | 0.06 | 0.20 | 30% |
| *Habitual disinhibition*^7^ | 0.07 | 0.53 | 0.16 | 0.04 | 0.20 | 19% |
| *Emotional disinhibition*^7^ | 0.07 | 0.40 | 0.17 | 0.03 | 0.20 | 14% |
| *Situational disinhibition*^7^ | 0.08 | 0.29 | 0.17 | 0.02 | 0.20 | 12% |
| Hunger^7^ | 0.11 | 0.23 | 0.17 | 0.03 | 0.20 | 13% |
| *External hunger*^7^ | 0.11 | 0.22 | 0.17 | 0.02 | 0.20 | 13% |
| *Internal hunger*^7^ | 0.09 | 0.18 | 0.18 | 0.02 | 0.20 | 8% |
| **ALSPAC** |  |  |  |  |  |  |
| Disinhibition^8^ | 0.15 | 0.49 | 0.16 | 0.07 | 0.23 | 31% |
| *Habitual disinhibition*^8^ | 0.12 | 0.48 | 0.17 | 0.06 | 0.23 | 26% |
| *Emotional disinhibition*^8^ | 0.10 | 0.34 | 0.20 | 0.03 | 0.23 | 14% |
| *Situational disinhibition*^8^ | 0.12 | 0.28 | 0.20 | 0.03 | 0.23 | 14% |
| Emotional overeating^8^ | 0.09 | 0.33 | 0.21 | 0.03 | 0.23 | 12% |
| Emotional undereating^8^ | -0.06 | -0.24 | 0.22 | 0.01 | 0.23 | 6% |
| Enjoyment of food^8^ | -0.03 | 0.08 | 0.24 | 0.00 | 0.23 | N/A^9^ |
| Food fussiness^8^ | 0.02 | 0.12 | 0.23 | 0.00 | 0.23 | 1% |
| Food responsiveness^8^ | 0.01 | 0.10 | 0.23 | 0.00 | 0.23 | 0% |
| Hunger^8^ | -0.07 | -0.12 | 0.23 | 0.01 | 0.23 | 4% |
| Satiety responsiveness^8^ | -0.02 | -0.15 | 0.23 | 0.00 | 0.23 | 1% |
| Slowness in eating^8^ | -0.03 | -0.15 | 0.23 | 0.00 | 0.23 | 2% |
| **Intermediate confounding^10^** |  |  |  |  |  |  |
| Depression^11^ | 0.06 | 0.24 | 0.17 | 0.01 | 0.19 | 5.3% |

ALSPAC, Avon Longitudinal Study of Parents and Children study; Std.β, standardised beta coefficient; GATE, Genetics of Appetite study

^1^Reliability of eating behaviour 0.8 (error due to variance fixed to 20%). Smoking, ethnicity, depression, anxiety, sex, age were controlled for in relationship a; ethnicity, depression, anxiety, sex and age controlled for in relationship b (**Supplementary Figure 1**).

^2^a represents the association between BMI-GRS and eating behaviour.

^3^b represents the association between the eating behaviour and BMI, adjusted for BMI-GRS.

^4^The direct effect (c’) is exposure of BMI-GRS on BMI while adjusting for the eating behaviour (the mediator).

^5^The indirect (or the mediation) effect is the product of a and b (a * b). In the indirect effect quantifies how much of the effect of the BMI-GRS std on BMI goes through, or is mediated by, the eating behaviour.

^6^The total effect is the sum of the direct and indirect effects of BMI-GRS std.

^7^GATE TFEQ n=1219, ^8^ALSPAC TFEQ and AEBQ items n=1468.

^9^Proportion mediated not shown due to inconsistent mediation (i.e. whereby the total effect is the sum of the (counteracting) direct and indirect effect, but the proportion mediated is a minus percentage and therefore illogical).

^10^Anxiety was not an intermediate mediator between BMI-GRS and BMI in GATE or ALSPAC (results not reported).

^11^Depression score was a part mediator in the GATE cohort only (pathway a: std. beta 0.06, 95%CI 0.02 to 0.10, pathway b: 0.24, 0.20 to 0.28, indirect effect 0.01, 0.004 to 0.02, direct effect 0.17, 0.14 to 0.21). Including eating behaviours as a confounder into the depression mediation model removed the mediation between BMI-GRS and BMI except for TFEQ-internal hunger. The percentage of the BMI-GRS to BMI effect mediated by depression score was similar in the sensitivity model without and with internal hunger (i.e. 5.3% versus 5.6%, respectively).

Table S5: pathway a and b, stratified by flexible and rigid restraint^1^

|  | a (Std.β, 95% CI)^2^ | | b (Std.β, 95% CI)^3^ | |
| --- | --- | --- | --- | --- |
| **GATE** | Low flexible restraint | High flexible restraint | Low flexible restraint | High flexible restraint |
| Disinhibition | 0.13 (0.08 to 0.18) | 0.07 (-0.002 to 0.13) | 0.52 (0.47 to 0.56) | 0.31 (0.25 to 0.38) |
| *Habitual disinhibition* | 0.12 (0.07 to 0.17) | 0.06 (-0.01 to 0.14) | 0.50 (0.45 to 0.55) | 0.30 (0.23 to 0.36) |
| *Emotional disinhibition* | 0.10 (0.05 to 0.16) | 0.03 (-0.04 to 0.10) | 0.40 (0.35 to 0.44) | 0.26 (0.19 to 0.32) |
| *Situational disinhibition* | 0.09 (0.04 to 0.14) | 0.05 (-0.02 to 0.12) | 0.33 (0.28 to 0.38) | 0.15 (0.08 to 0.22) |
| Hunger | 0.13 (0.07 to 0.18) | 0.05 (-0.02 to 0.11) | 0.24 (0.19 to 0.29) | 0.17 (0.10 to 0.24) |
| *External hunger* | 0.11 (0.06 to 0.16) | 0.06 (-0.01 to 0.13) | 0.26 (0.21 to 0.31) | 0.13 (0.06 to 0.20) |
| *Internal hunger* | 0.10 (0.05 to 0.15) | 0.03 (-0.04 to 0.10) | 0.16 (0.11 to 0.21) | 0.16 (0.09 to 0.23) |
| **ALSPAC** |  |  |  |  |
| Disinhibition | 0.18 (0.13 to 0.23) | 0.01 (-0.09 to 0.11) | 0.46 (0.41 to 0.51) | 0.24 (0.15 to 0.32) |
| *Habitual disinhibition* | 0.15 (0.10 to 0.20) | 0.01 (-0.09 to 0.11) | 0.48 (0.42 to 0.53) | 0.26 (0.17 to 0.34) |
| *Emotional disinhibition* | 0.12 (0.06 to 0.17) | 0.03 (-0.06 to 0.13) | 0.31 (0.25 to 0.36) | 0.20 (0.11 to 0.29) |
| *Situational disinhibition* | 0.15 (0.09 to 0.20) | 0.002 (-0.10 to 0.10) | 0.29 (0.23 to 0.34) | 0.10 (0.02 to 0.19) |
| Emotional overeating | 0.09 (0.04 to 0.15) | 0.04 (-0.06 to 0.13) | 0.28 (0.23 to 0.33) | 0.24 (0.15 to 0.32) |
| Emotional undereating | -0.08 (-0.13 to -0.02) | 0.03 (-0.06 to 0.12) | -0.19 (-0.25 to -0.14) | -0.18 (-0.27 to -0.09) |
| Enjoyment of food | 0.01 (-0.04 to 0.06) | -0.07 (-0.18 to 0.04) | 0.05 (-0.01 to 0.11) | 0.06 (-0.02 to 0.13) |
| Food fussiness | 0.004 (-0.05 to 0.06) | 0.04 (-0.05 to 0.14) | 0.11 (0.05 to 0.16) | 0.09 (-0.004 to 0.17) |
| Food responsiveness | 0.03 (-0.03 to 0.08) | -0.05 (-0.14 to 0.05) | 0.11 (0.05 to 0.16) | 0.01 (-0.08 to 0.09) |
| Hunger | -0.06 (-0.11 to -0.004) | -0.07 (-0.16 to 0.02) | -0.11 (-0.16 to -0.05) | -0.04 (-0.14 to 0.05) |
| Satiety responsiveness | -0.03 (-0.09 to 0.02) | -0.03 (-0.12 to 0.07) | -0.12 (-0.18 to -0.07) | -0.09 (-0.18 to 0.00) |
| Slowness in eating | -0.04 (-0.09 to 0.01) | -0.01 (-0.10 to 0.08) | -0.13 (-0.18 to -0.07) | -0.07 (-0.16 to 0.02) |
| **GATE** | Low rigid restraint | High rigid restraint | Low rigid restraint | High rigid restraint |
| Disinhibition | 0.12 (0.07 to 0.16) | 0.07 (-0.01 to 0.15) | 0.12 (0.07 to 0.16) | 0.37 (0.28 to 0.45) |
| *Habitual disinhibition* | 0.10 (0.06 to 0.15) | 0.08 (-0.004 to 0.16) | 0.10 (0.06 to 0.15) | 0.27 (0.20 to 0.35) |
| *Emotional disinhibition* | 0.08 (0.03 to 0.12) | 0.07 (-0.004 to 0.15) | 0.08 (0.03 to 0.12) | 0.29 (0.21 to 0.37) |
| *Situational disinhibition* | 0.09 (0.04 to 0.13) | 0.04 (-0.04 to 0.11) | 0.09 (0.04 to 0.13) | 0.21 (0.13 to 0.30) |
| Hunger | 0.1 (0.05 to 0.15) | 0.08 (0.004 to 0.16) | 0.10 (0.05 to 0.15) | 0.18 (0.09 to 0.26) |
| *External hunger* | 0.1 (0.05 to 0.15) | 0.07 (-0.01 to 0.15) | 0.10 (0.05 to 0.15) | 0.17 (0.09 to 0.25) |
| *Internal hunger* | 0.07 (0.02 to 0.12) | 0.08 (0.001 to 0.16) | 0.07 (0.02 to 0.12) | 0.16 (0.07 to 0.24) |
| **ALSPAC** |  |  |  |  |
| Disinhibition | 0.14 (0.09 to 0.19) | 0.10 (0.01 to 0.20) | 0.49 (0.44 to 0.55) | 0.25 (0.17 to 0.33) |
| *Habitual disinhibition* | 0.11 (0.06 to 0.15) | 0.11 (0.01 to 0.21) | 0.54 (0.48 to 0.60) | 0.26 (0.19 to 0.34) |
| *Emotional disinhibition* | 0.11 (0.06 to 0.16) | 0.04 (-0.05 to 0.13) | 0.32 (0.26 to 0.37) | 0.19 (0.10 to 0.27) |
| *Situational disinhibition* | 0.11 (0.06 to 0.16) | 0.08 (-0.01 to 0.17) | 0.28 (0.23 to 0.34) | 0.13 (0.05 to 0.22) |
| Emotional overeating | 0.06 (0.01 to 0.12) | 0.11 (0.02 to 0.21) | 0.30 (0.25 to 0.35) | 0.20 (0.13 to 0.28) |
| Emotional undereating | -0.06 (-0.11 to -0.002) | -0.04 (-0.13 to 0.06) | -0.21 (-0.26 to -0.15) | -0.17 (-0.25 to -0.09) |
| Enjoyment of food | -0.03 (-0.08 to 0.03) | 0.01 (-0.08 to 0.10) | 0.05 (-0.003 to 0.11) | 0.06 (-0.03 to 0.14) |
| Food fussiness | 0.01 (-0.04 to 0.07) | 0.03 (-0.05 to 0.12) | 0.10 (0.04 to 0.15) | 0.13 (0.04 to 0.21) |
| Food responsiveness | -0.01 (-0.06 to 0.05) | 0.02 (-0.07 to 0.12) | 0.10 (0.05 to 0.16) | 0.02 (-0.06 to 0.11) |
| Hunger | -0.07 (-0.12 to -0.01) | -0.05 (-0.14 to 0.04) | -0.10 (-0.16 to -0.05) | -0.07 (-0.16 to 0.01) |
| Satiety responsiveness | -0.01 (-0.06 to 0.04) | -0.06 (-0.15 to 0.03) | -0.14 (-0.19 to -0.08) | -0.06 (-0.15 to 0.03) |
| Slowness in eating | -0.03 (-0.08 to 0.02) | -0.03 (-0.12 to 0.06) | -0.15 (-0.20 to -0.09) | -0.03 (-0.12 to 0.06) |

AEBQ, Adult Eating Behaviour Questionnaire; ALSPAC, Avon Longitudinal Study of Parents and Children; Std.β, standardised beta coefficient; BMI, BMI, body-mass-index; BMI-GRS, body-mass-index genetic risk score; GATE, Genetics of Appetite; TFEQ-51, Three-factor Eating Questionnaire-51; 95% CI, confidence interval

^1^Analyses shown here were stratified into ‘high’ and ‘low’ flexible restraint (a score of ≤3 was low restraint, >3 was high restraint (Aurelie et al., 2012; Kruger et al., 2016) to illustrate the effects of flexible and rigid restraint on the mediation pathways in a binary fashion. Models were adjusted for age and sex. Direct, indirect and total effects of BMI-GRS available in **Table 2** and **3**

^2^a represents the association between BMI-GRS and eating behaviour.

^3^b represents the association between the eating behaviour and BMI, adjusted for BMI-GRS.
